# Dimerization of Human Angiogenin and of Variants Involved in Neurodegenerative Diseases

**DOI:** 10.3390/ijms221810068

**Published:** 2021-09-17

**Authors:** Sabrina Fasoli, Ilaria Bettin, Riccardo Montioli, Andrea Fagagnini, Daniele Peterle, Douglas V. Laurents, Giovanni Gotte

**Affiliations:** 1 Department of Neuroscience, Biomedicine, and Movement Sciences, Biological Chemistry Section, University of Verona, Strada Le Grazie 8, I-37134 Verona, Italy; sabrina.fasoli@univr.it (S.F.); ilaria.bettin@univr.it (I.B.); riccardo.montioli@univr.it (R.M.); andrea.fagagnini@hotmail.it (A.F.); 2 Instituto de Química Física “Rocasolano”, Consejo Superior de Investigaciones Científicas, Serrano 119, E-28006 Madrid, Spain; dlaurents@iqfr.csic.es; 3 Department of Pharmaceutical Sciences, University of Padua, Via Marzolo 5, I-35131 Padua, Italy; daniele.peterle@unipd.it

**Keywords:** human angiogenin, ribonuclease, protein oligomerization, 3D domain swapping, amyotrophic lateral sclerosis (ALS), Parkinson’s disease (PD)

## Abstract

Human Angiogenin (hANG, or ANG, 14.1 kDa) promotes vessel formation and is also called RNase 5 because it is included in the pancreatic-type ribonuclease (pt-RNase) super-family. Although low, its ribonucleolytic activity is crucial for angiogenesis in tumor tissues but also in the physiological development of the Central Nervous System (CNS) neuronal progenitors. Nevertheless, some ANG variants are involved in both neurodegenerative Parkinson disease (PD) and Amyotrophic Lateral Sclerosis (ALS). Notably, some pt-RNases acquire new biological functions upon oligomerization. Considering neurodegenerative diseases correlation with massive protein aggregation, we analyzed the aggregation propensity of ANG and of three of its pathogenic variants, namely H13A, S28N, and R121C. We found no massive aggregation, but wt-ANG, as well as S28N and R121C variants, can form an enzymatically active dimer, which is called ANG-D. By contrast, the enzymatically inactive H13A-ANG does not dimerize. Corroborated by a specific cross-linking analysis and by the behavior of H13A-ANG that in turn lacks one of the two His active site residues necessary for pt-RNases to self-associate through the three-dimensional domain swapping (3D-DS), we demonstrate that ANG actually dimerizes through 3D-DS. Then, we deduce by size exclusion chromatography (SEC) and modeling that ANG-D forms through the swapping of ANG N-termini. In light of these novelties, we can expect future investigations to unveil other ANG determinants possibly related with the onset and/or development of neurodegenerative pathologies.

## 1. Introduction

**Figure 1:**
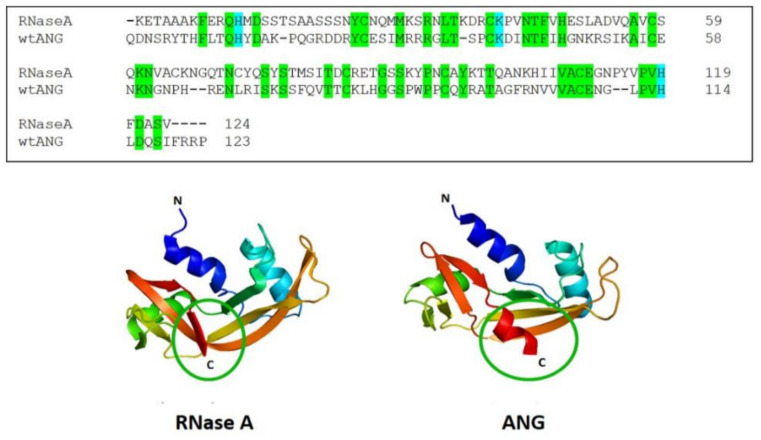
RNase A and ANG. Primary (**upper panel**) and 3D structures (**lower panel**) of RNase A and ANG (5RSA.pdb and 1ANG.pdb, respectively). In the sequence, the common residues are highlighted in green, while the catalytic triad is in light blue. In the lower panel, the different regions, especially the helices, are shown with different colours, while the different conformations of the C-termini of ANG are circled.

Human angiogenin (hANG, or ANG) is a 123 residue 14.1 kDa protein first isolated from human adenocarcinoma cell-conditioned media [1,2]. ANG is a secreted growth factor present in normal human tissues and plasma, or amniotic fluid [3,4], so called because it promotes, together with other proteins, chemokines, factors, and cells [5], the neo-formation of vessels [1,6]. ANG is also known as ribonuclease 5 (RNase 5) [7] because it displays the typical size and fold of the secretory pancreatic-type (pt)-RNases [8], and its catalytic triad is composed of His13, Lys40, and His114 [9,10], corresponding to the H12, K41, and H119 residues in bovine pancreatic RNase A, the super-family archetype [11]. ANG and RNase A share only 33% sequence identity and 65% sequence similarity [1], and ANG lacks the C65-C72 short disulfide but possesses an extra 3_10_-helix at its C-terminus [8]. Nevertheless, their tertiary structures are quite similar, as shown in Figure 1 [12].

ANG ribonucleolytic activity is 10^4^–10^6^ lower than that of RNase A [13], but both follow the same catalytic mechanism [10,14]. One of the reported reasons for this low activity is the presence of the Q117 residue near the C-terminus, which obstructs the pyrimidine binding site [15]. Nevertheless, ANG is active enough vs. rRNAs [16], tRNAs [17], or vs. particular oligonucleotides as well [18]. Moreover, it exerts many important biological functions beyond angiogenesis, such as inducing wound healing [19], promoting reproduction [20], and neuroprotective effects [21]; all these functions require ANG enzymatic activity [22]. Cytoplasmic ANG is also known to be relocated in stress granules, to potentiate their stress-induced formation by the producing of tiRNA [23], and to regulate cell growth and survival [24]. The ANG cell internalization pathway is not well known yet [25], but some evidence suggests that it requires a receptor-mediated endocytosis followed by nuclear localization via a transport process that is lysosome- and dynamin-independent [26,27]. This transport involves the so-called ANG nuclear localization signal (NLS) whose sequence is 30-MRRRG-34 [28]. Alternatively, ANG can pass through the nuclear pores to activate the transcription of angiogenic or epidermal growth factors [29], since it is definitely smaller than the pores’ 50 kDa size limit [30].

Despite expressing many positive functions, ANG can favour the growth of solid tumors by inducing neovascularization around the malignant tissue mass [16], while some ANG mutants are implicated in neurodegenerative diseases, because their dysfunction often leads to motor neurons hypoxia and death [31]. Many variants arising from pathogenic mutations influencing the ANG structure; substrate binding and therefore ribonucleolytic [32] or biological activities [32,33,34,35] have been found in the recent past. Importantly, mutations affecting ANG ability to be translocated into the nucleus [36] have been detected in various cohorts of amyotrophic lateral sclerosis (ALS) and/or Parkinson’s disease (PD) patients [37,38]. In addition, we recently reported the effects of two ANG pathogenic variants on the tRNA processing and G-quadruplex formation [32].

Importantly, some members of the pt-RNase superfamily, above all the archetype RNase A, are able to form domain-swapped dimers or larger oligomers [39,40,41] through the three-dimensional domain swapping (3D-DS) mechanism [42]. These oligomers display biological properties absent in the native monomer or modulate pre-existing activities [25]. Indeed, it is known that protein aggregation can promote neurodegenerative diseases. Therefore, we studied here the self-association propensity of ANG and of three of its pathogenic variants found in amyotrophic lateral sclerosis (ALS) and Parkinson’s Disease (PD) patients, namely H13A-ANG, S28N-ANG, and R121C-ANG. We chose the first mutant because it is inactive, lacking one of the three catalytic residues [32]; the second had been envisaged in a previous report to be possibly inclined to dimerize [43], while the third displays a higher RNase activity than wt-ANG [35]. This has been ascribed to a decreased steric hindrance affecting the catalytic site cleft caused by the R > C mutation [35]. Moreover, we aimed to investigate if a free cysteine, absent in the wt, may affect the dimerization tendency of ANG, such as, for example, it occurs for bovine seminal RNase [44] or for the signal transducer and activator of transcription 3 (STAT3) [45].

The data we collected demonstrate that wt-ANG, as well as S28N-ANG and R121C-ANG, can be induced to dimerize following the same 3D-DS mechanism of other pt-RNases [39,40,41], while the H13A mutant does not. Interestingly, since the dimerization tendency had been hypothesized in the past only for S28N-ANG [43], future studies may investigate if this event has implications for ANG’s intracellular distribution and functions and for the related pathologies.

## 2. Results

### 2.1. Production, Purification, and Analysis of the ANG Variants

**Figure 2:**
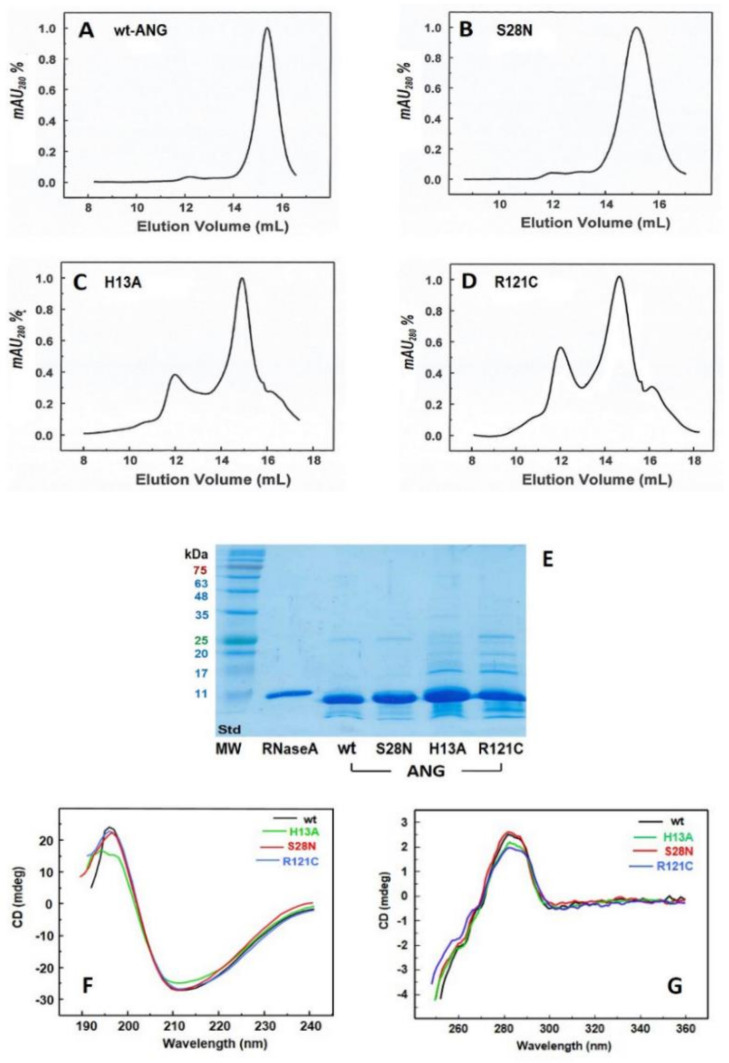
Purification and analysis of the ANG variants produced. (**A**–**D**): SEC patterns of the last purification step performed upon the expression of each ANG variant. Chromatography was performed with a Superdex 75 HR 10/300 column equilibrated with 0.1M Tris-HCl, pH 7.4. The main peak of each variant, which elutes around 15 mL, has been desalted, aliquoted, lyophilized from ddH_2_O, and stored until further analysis; (**E**): 15% acrylamide SDS-PAGE of the main peak recovered from SEC purification of each ANG variant indicated, with the colored molecular weight (MW) standards running in the first lane and labeled with same colors. Gel was run at 200 mV for 90 min. (**F**,**G**): Far- and near-UV CD spectra of the four ANG variants.

**Figure 3:**
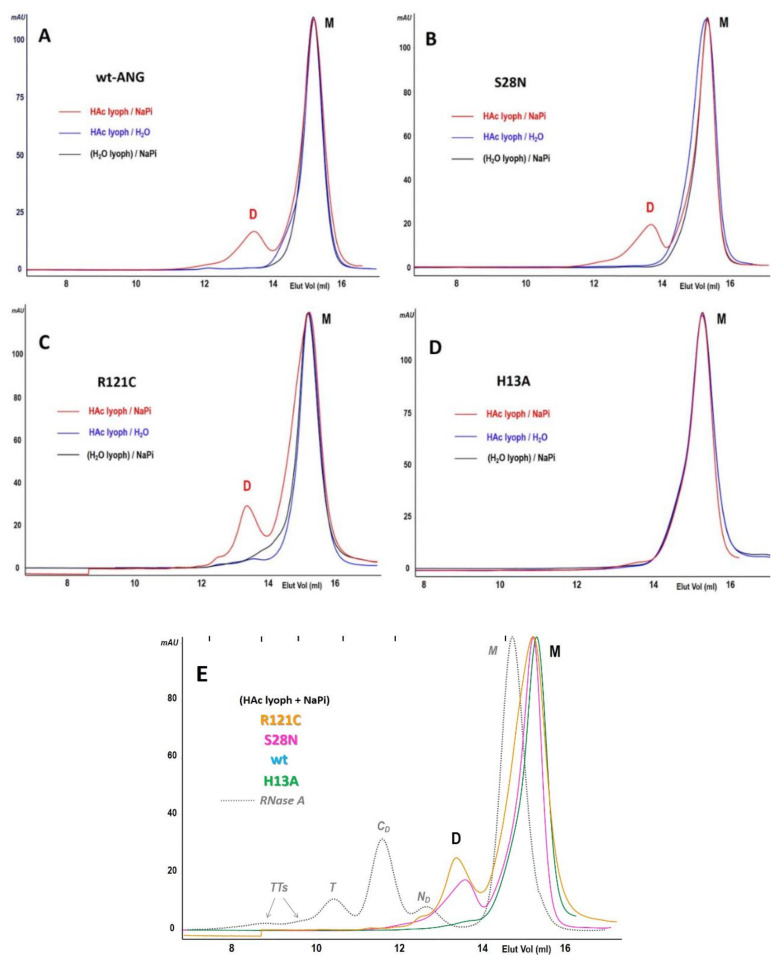
SEC profiles of the dimerization of three ANG variants. (**A**–**D**): Analysis of ANG variants WT, S28N, R121C, and H13A-ANG after direct solubilization in NaPi, pH 6.7 (black curves), or after lyophilization from 40% HAc and solubilization in H_2_O (blue curves) or in 0.40 M NaPi, pH 6.7 (red curves). Each chromatogram is representative of five (black and blue curves) or eight different tests (red curve). M: monomer, D: dimer.; (**E**): Superposition of the patterns obtained with the three ANG variants after lyophilization from 40% HAc and solubilization in NaPi, compared with the known profile of RNase A. M: ANG monomer, D: ANG dimer, *N_D_* or *C_D_*: N-swapped or C-swapped RNase A dimer(s) of RNase A, *T* and *TTs*: RNase A domain-swapped trimer and tetramers [39]. The positions of the MW standards (Supplementary Figure S2) are indicated with black lines in the upper part of the panel. All A-E patterns were obtained with a Superdex 75 HR 10/300 Increase column equilibrated with 0.40 M NaPi, pH 6.7, with a 0.20–0.25 mL/min flow rate, and normalized to the monomer area with the Unicorn 5.01 software.

The expression of wt-ANG as well as the H13A S28N and R121C-ANG variants was monitored step by step with SDS-PAGE (not shown). Afterwards, a final SEC purification of the protein was performed with a Superdex 75 HR 10/300 column, whose chromatograms are shown in Figure 2A–D. The elution volume of ANG fell around 15 mL, which is similar to the value (14.2 mL) of monomeric RNase A. We obtained almost pure wt- and S28N-ANG, since they both showed only one peak corresponding to the desired protein species. By contrast, the H13A- and R121C-ANG chromatograms showed a less abundant peak around 12 mL, which was eluted before the ANG species (Figure 2C,D). The SDS-PAGE analysis showed that the mean peak of H13A and R121C variants were only slightly less pure than the wt and the S28N-ANG (Figure 2E). However, we performed another SEC purification (not shown) to eliminate these impurity traces (see later in Figure 3, black curves). Then, we measured, with mass spectrometry (MS), the experimental MW of the species eluted in the main peak of each variant: indeed, the values found overlap the expected calculated values (Table 1, Supplementary Figure S1A–E). In contrast, the two peaks preceding H13A and R121C monomers, which eluted at about 12 mL (Figure 2C,D), did not show in MS MW values compatible with multiples of each corresponding ANG monomer (not shown).

Then, we analyzed the SEC-purified proteins with circular dichroism (CD). Figure 2F,G show no significant differences within far- and near-UV CD spectra of wt-ANG, S28N, and R121C-ANG. This suggests that neither the S28N nor the R121C substitutions affect the protein secondary structure. The near-UV spectra indicate that the conformation of the regions surrounding the aromatic residues reside are not perturbed by the mutation, suggesting that S28N-ANG and R121C-ANG variants retain the same global tertiary structure of the wt. In contrast, the far-UV spectrum of H13A-ANG displays some slight differences with respect to the other two ANG variants, especially between 190 and 200 nm, suggesting that a perturbation occurs in the first α-helix where the mutation is located, or even in the residues near H13, some of which belong to a β-sheet. However, since both the α-helix and β-sheet produce a CD maximum between 190 and 200 nm, it is difficult to rule out which protein regions are actually affected.

### 2.2. WT, S28N, and R121C-ANG Dimerize If Dissolved in NaPi Buffer after Lyophilization from HAc Solutions, while the H13A Variant Does Not

Aliquots of each ANG species were lyophilized after dissolving them in 40% acetic acid (HAc) at a concentration of approximately 25 mg/mL [46]. In order to detect the possible formation of dimer(s) or oligomers, the resulting powder deriving from each ANG variant was solubilized in 0.40 M NaPi, pH 6.7 [40], and the solution mixture was analyzed with a Superdex 75 Increase HR10/300 SEC column (Figure 3A–D, red curves). The WT, as well as the S28N and R121C variants, but not the H13A mutant, showed a peak eluting at about 13.5 mL, i.e., before the monomer, that eluted at about 15.3 mL. Although called D and M, respectively for putative dimer and monomer, both peaks of each variant correspond to MWs that are definitely lower than the expected values (Table 1) based on the column calibration curve (Supplementary Figure S2). Nevertheless, the D/M MW ratio calculated from SEC is close to two. This suggests that the peak eluting around 13.5 mL is an ANG dimer, so we tentatively call it ANG-D. Incidentally, the chromatographic behavior of ANG-D resembles those of ONC-D and the RNase A N-dimer forming by the same HAc lyophilization/NaPi solubilization procedure (dotted curve of Figure 3E) [39,41].

S28N-ANG shows a higher dimerization propensity than wt-ANG, and the R121C mutant’s dimerization yield is almost double that of wt ANG-D yield (Figure 3E), as confirmed by up to eight tests performed with each variant, whose average yield values are reported in Table 1: the R121C mutant approaches 18% dimer yield, while S28N is about 15% and for wt-ANG only about 10% of the total species eluted are dimeric. The higher yield of R121C and S28N dimers vs. wt-ANG are statistically significant. Traces of an apparently larger species can be visible in both variants’ patterns, but no precise quantification could be performed, and their yields are not reported in Table 1.

Other HAc-lyophilized samples of all ANG variants were dissolved in ddH_2_O lacking NaPi buffer. The blue curves reported in Figure 3A–D show that none of the variants form any detectable dimer. This behavior parallels those of RNase A and of other pt-RNases that do not form non-covalent RNase oligomers through 3D-DS if phosphate is absent in the solubilization buffer: indeed, a phosphate electrostatic bridge connecting the two active site His residues located in the two N- and C-termini of the enzyme is necessary for RNases’ oligomers to be stabilized [47].

In addition, we directly solubilized with 0.40 M NaPi buffer other WT-, S28N, or R121C-ANG samples without previously subjecting them to HAc lyophilization: again, the corresponding SEC patterns did not show any species different from the monomer (Figure 3A–D, black curves), confirming as well the purity of each ANG variant used and already detected with MS. Therefore, the results obtained confirm that only the combination of acidic lyophilization followed by NaPi solubilization can induce ANG dimerization, which is in accordance with other pt-RNases that oligomerize through 3D-DS [40,41,48].

We mentioned before that the dimerization tendency of the enzymatically inactive H13A-ANG was also analyzed upon 40% HAc lyophilization followed by NaPi solubilization, but the SEC analysis showed no dimer at all (red curve of Figure 3D). Obviously, no H13A-ANG dimer formed directly from ddH_2_O lyophilization plus NaPi solubilization, or even after HAc lyophilization followed by ddH_2_O solubilization (Figure 3C, black and blue curves, respectively). Hence, the inability of H13A-ANG to dimerize parallels the results obtained with other pt-RNases lacking one (or both) His active site residue(s) [41]. These findings, and those described in the preceding two paragraphs, strongly suggest that the other ANG variants dimerize through the 3D-DS mechanism and that the dimers are stabilized by a H13–phosphate–H114 salt bridge [47].

### 2.3. Analysis of the ANG Dimerization Mechanism with Divinyl Sulfone (DVS)

**Figure 4:**
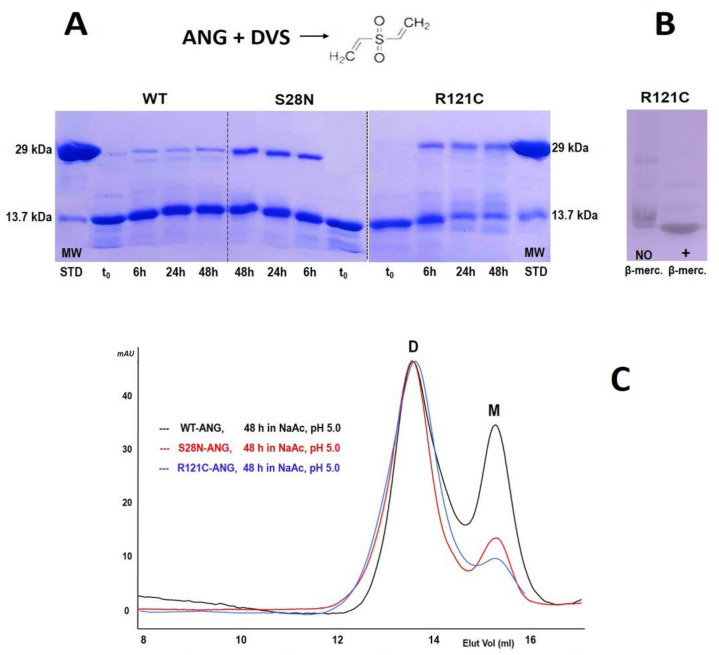
Analysis of ANG-D. (**A**): 15% acrylamide SDS-PAGE of the cross-linking reaction of ANG-D of WT, S28N, and R121C-ANG variants performed with divinyl sulfone (DVS), whose structure is reported in the panel; (**B**): 15% acrylamide SDS-PAGE of R121C-ANG under reducing or non-reducing conditions. Gels were run in both panels at 200 V for 80 min; (**C**): SEC analysis of ANG-D upon its incubation at RT for 48 h in 0.1 M sodium acetate/HAc buffer, pH 5.0. M: monomer, D: dimer. All chromatograms have been registered with a 0.20–0.25 mL/min flow rate in the same Superdex 75 HR 10/300 Increase column equilibrated with 0.20 M NaPi, pH 6.7.

In order to gain further insight into the mechanism of ANG dimerization, we induced the dimers to react with divinyl sulfone (DVS), which is a short cross-linker (Figure 4). DVS is useful to assess if an RNase undergoes 3D-DS: indeed, if ANG dimerizes through 3D-DS, DVS should cross-link the two active site H13–H114 residues located in the two N- and C-termini of ANG, respectively [48]. Thus, the reaction would covalently stabilize ANG-D and make it resistant to SDS-PAGE denaturing conditions. Indeed, as reported in Figure 4A, the SDS-PAGE of all dimers following DVS incubation showed the presence of some covalently stabilized dimer. The dimeric nature (MW ≈ 28.3 kDa) of these ANG species is confirmed by their electrophoretic mobility being identical to the 29 kDa MW standard carbonic anhydrase. The DVS reaction did not reach completion, with the amount of covalently stabilized dimer reaching a plateau after about 24 h incubation with all ANG variants. We recall here that the DVS reaction never reaches completion with the oligomers of other pt-RNases, such as RNase A [49,50,51], bovine seminal RNase [40,48], or also onconase [41]. The conditions required for a productive reaction are pH 5.0 in acetate/HAc and 30 °C [48], although here, we kept the mixture at room temperature (RT) to possibly diminish the tendency toward a contemporary dissociation to monomer during the cross-linking reaction [49,50,51].

Hence, we analyzed the stability of the three ANG-D after 48 h incubation in HAc/Acetate, pH 5.0, at RT. Figure 4C shows that the dimer formed by wt-ANG is less stable than those of S28N and R121C, which is in line with the yields of dimers visible in the SDS-PAGE performed after the stabilizing DVS reaction and shown in panel A. We also underline that ANG possesses more His residues than other RNases, such as for example RNase A (Figure 1, upper panel), and some of them are located at distances compatible for competing with the two active site His belonging to two different subunits placed close together following 3D-DS (Supplementary Figure S3). The formation of a DVS cross-link between one of these other His and an active site His in the 3D-DS dimer would result in a species unable to resist monomerization during SDS-PAGE. These events, and a contemporary partial monomerization, could account for the observation of monomeric species. Finally, we also tested if the highest ANG-D yield obtained with the R121C mutant could result from the formation of a C121A-C121B disulfide, with A and B representing the two ANG coupling monomers. Instead, Figure 4B shows the R121C ANG-D does not resist under denaturation conditions even when the β-mercaptoethanol reducing agent is absent. This indicates the mentioned inter-subunit disulfide does not form and also the R121C variant dimerizes only through 3D-DS.

### 2.4. ANG-D Is Remarkably Stable in NaPi Buffer at 4 °C

**Figure 5:**
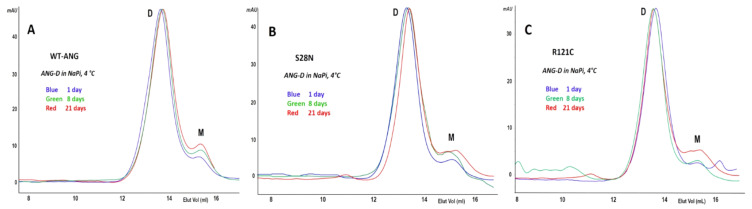
SEC analysis of the intrinsic stability of ANG-D. (**A**,**B**): wt and S28N-ANG-D after 2 (blue), 10 (green), and 21 (red) days of storage at 0.5 mg/mL in 0.4 M NaPi, pH 6.7, at 4 °C; (**C**): wt- and S28N-ANG-D (blue and red, respectively) after 48 h of storage at RT in 0.10 M sodium acetate, pH 5.0. M: monomer, D: dimer.

The ANG-D formed by wt, S28N, or R121C-ANG were separately collected and stored at 4 °C at a concentration of about 0.10–0.15 mg/mL in 0.20 M NaPi, pH 6.7. The non-denaturing cathodic PAGE did not provide an insightful electrophoretic mobility difference between ANG and ANG-D, even when it was run with different acrylamide percentages (Supplementary Figure S4). Therefore, we used SEC to monitor the dimers’ resistance to dissociation and regression to monomer after incubation at 4 °C for times ranging from one to 21 days. The SEC patterns reported in Figure 5A–C show that all ANG-D variants undergo little regression to monomer, hence indicating once again that phosphate and low temperatures allow them to resist dissociation for a long time.

### 2.5. All ANG-D Variants Retain Ribonucleolytic Activity

The enzymatic activity values of all ANG variants and of their relative dimers on 6-FAM-dArU(dA)_2_-BHQ-1 substrates are reported in Table 1: all ANG-D retain a high percentage of activity with respect to each corresponding native monomer. This is compatible with the 3D-DS nature of ANG-D, since 3D-DS permits the reconstitution of the so-called functional unit (FU), as described by Liu and Eisenberg [52]. Interestingly, the S28N-ANG monomer is not excessively less active than the wt monomer. This suggests that this mutation impacts activity on a tetranucleotide less than it does on a tRNA, as reported previously [34]. In terms of the specific activity per mole, Table 1 shows that all ANG-D are less active than their ANG monomers, suggesting that the dimer structure can permit only one FU out of the two to be active. This could be due to a reciprocal steric hindrance occurring within two substrate molecules. This takes place due to the presence of two enzyme FUs that in the dimer are very close to one another. Similar arguments have been advanced in the past and more recently to justify the activity of the RNase A oligomers vs. yeast RNA [51,53]. Logically, the H13A variant displayed no activity because the mutation affects, directly and negatively, the enzyme catalytic triad.

### 2.6. Molecular Modeling Supports the Hypothesis That ANG-D Forms through the Swapping of ANG N-Termini

**Figure 6:**
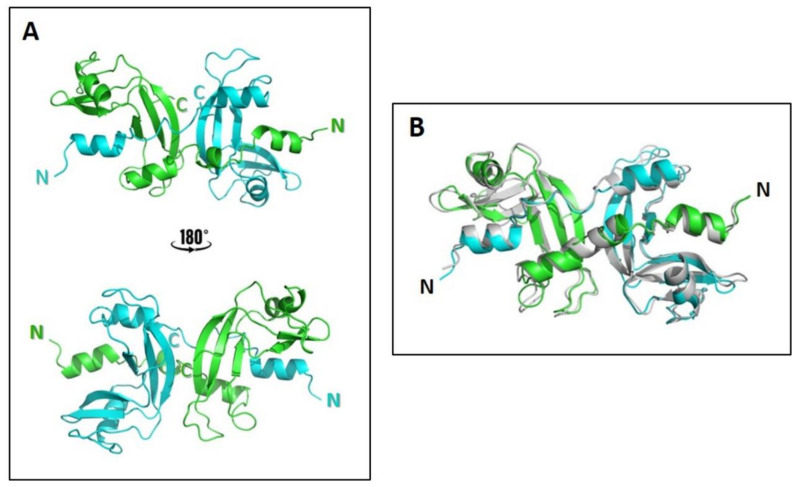
Molecular modeling of ANG-N_D_. (**A**): Cartoon representation of the ANG-N_D_ modeled structure minimized with the Opler force field. The model is visible from two opposite angles with the two protomers colored in green and cyan, respectively; (**B**): superimposition of the ANG-N_D_ model (green–cyan) with the RNase A-N_D_ crystal structure (gray, pdb 1A2W [56]). Images were rendered with the PyMol v0.99 software (Schrödinger).

The experimental data strongly suggest that the fast-eluting SEC peak is a domain-swapped ANG-D. To corroborate these results and get insight into its quaternary structure, we modeled the dimer starting from the crystal structures of the two N- and C-termini domain-swapped dimers, N_D_ and C_D_, of RNase A [39,55,56]. The ANG-N_D_ model reported in Figure 6 was constructed by homology modeling and refined by energy minimization. Whereas RNase A and ANG share only 33% sequence identity, the predicted secondary structure of ANG-N_D_ model is very close to that of RNase A-N_D_. Larger differences are visible in the C-terminal region, where ANG in endowed with a four-residue elongation (120-FRRP-123) with respect to RNase A, as demonstrated by the sequence alignment reported in Figure 1 and in the Supplementary Figure S5. Therefore, even though we built a model based on RNase A-C_D_, this model could not be refined. In particular, the absence of ANG residues corresponding to the Pro114-Tyr115 segment in RNase A interrupts the C-terminal arm in the obtained ANG-C_D_ model (Supplementary Figure S5). Indeed, in RNase A-C_D_, the latter segment represents a key β-strand portion connecting the two subunits and forming the open interface [42] that contributes to stabilizing the dimer [57]. Hence, it is reasonable to suggest that the lack of the Pro114-Tyr115 residues, together with the presence of the aforementioned C-terminal tail 120-FRRP-123, could lower the possibility of the C-terminal domains exchanging between ANG monomers. Overall, and finally, modeling suggests that ANG-D forms more probably through the swapping of the N-termini of the protein rather than via a C-terminus swapping. Whereas the N-termini swapping model built is very likely and consistent with all the results, we cannot exclude the possibility that the real structure of ANG-N_D_ could be different. By the way, we recall that the RNase A C-swapped cyclic trimer’s crystal structure displayed different reciprocal subunit orientations with respect to a C-swapped structural model [55].

## 3. Discussion

In this study, the self-association tendency of human angiogenin (ANG) has been analyzed for the first time. Indeed, since some variants of this protein are involved in neurodegenerative diseases, such as ALS and PD, and taking into account that protein massive aggregation and fibrillization are hallmarks of many neurodegenerative diseases, we investigated if ANG might undergo significant aggregation events. To do so, we produced, characterized, and analyzed the behavior of wt-ANG, as well as of its H13A, S28N, and R121C pathogenic mutants (Figure 2A–E). The far-UV CD spectra of S28N and R121C-ANG showed no differences with respect to that of wt, while the H13A variant showed some small variations indicating only a slight perturbation affecting the secondary structure (Figure 2F). Although it is not easy to predict which are the specific secondary structure elements affected by the H13A substitution, we could speculate that the alteration of a possible interaction involving the His13 of the N-terminal α-helix and the side chain of Asn43, which was included in the 41–47 β-strand, could affect the H13A mutant. The near-UV CD spectra indicate instead that none of the mutants undergo dramatic changes in their tertiary structures (Figure 2G).

Next, we first analyzed the aggregation propensity of wild-type ANG and of S28N and R121G-ANG variants. We chose the first pathogenic mutant because it had been previously considered to be prone to dimerize [43], and the S28N mutation is known to make the 16–20 loop connecting the N-terminus with the protein core more flexible than in wt-ANG [34]. Regarding the R121C variant, it was chosen because, despite being pathogenic, it is enzymatically more active than wt, and above all because the mutation affects the C-terminus of the enzyme [35].

The relative SEC patterns showed that upon lyophilization from 40% HAc solutions and powder solubilization in 0.4 M NaPi, pH 6.7, a peak eluted before the monomer (M) for wt and both S28N and R121C-ANG (Figure 3A–C). The calculated MW of both peaks are definitely lower than the expected ones (Table 1), which is probably due to non-specific interactions of ANG with the SEC matrix, and this apparently strange behavior somehow resembles the electrophoretic smear of ANG species in native cathodic PAGE (Supplementary Figure S4). However, considering that MS analysis confirms that the MW of each ANG variant overlaps the theoretical ones (Table 1, Supplementary Figure S1), that the calculated MW of the peak preceding the ANG monomer almost doubles the one of the latter (Table 1), and that DVS covalent stabilization showed species with electrophoretic mobility identical to carbonic anhydrase standard (MW = 29 kDa), we conclude that all these peaks, eluting at about 13.5 mL, correspond to a dimer (28.3 kDa, ANG-D). Furthermore, the S28N variant and especially R121C showed a higher tendency to dimerize than the wt (Table 1). Concerning S28N-ANG, the flexibility variation of the 16–20 loop connecting the N-terminus to the protein core and its increased solvent accessibility induced by the mutation, as highlighted by Acharya et al. in [34], may explain this result. As for R121C-ANG, the mutation affects the C-terminal 3_10_ helix [8], with the side chain becoming less bulky than in the wt [35]. Considering that the dimerization of some pt-RNases through N- or C-termini is certainly affected also by the nature and behavior of the opposite terminus [58,59], the lower hindrance ascribable to the R121C mutation may favor ANG dimerization through 3D-DS, which in turn almost doubles the ANG-D yield of the wt (Table 1). The SDS-PAGE analysis of the R121C dimer, under reducing or non-reducing conditions, indicates that the formation of this dimer is not driven by a disulfide involving the R121C residue of both monomers (Figure 4B).

In order to precisely unveil the mechanism of ANG dimerization, we used DVS to cross-link the H13 and H114 active site residues of each dimer subunit [48], and thereby corroborated that all the ANG variants formed the dimer through 3D-DS (Figure 4A) [41,55,56,60]. Indeed, although the reaction did not induce a dimer yield increase after 24 h incubation, the difference with the t_0_ scenario is clear. The impossibility of obtaining a higher dimer yield can be due to a concomitant dissociation of the dimer during the reaction (Figure 4C). However, we must recall and emphasize that the stability of the pt-RNase dimers or oligomers characterized to date depends on several parameters, such as temperature, oligomer concentration, pH, buffer type and concentration, or denaturants [40,41,47,51,58,60,61,62], but in our case, the yield of the DVS-covalently stabilized ANG-D (Figure 4A) reflects its stability in the pH 5 buffer used for the reaction (Figure 4C). Therefore, it is clear that ANG certainly dimerizes through 3D-DS.

The molecular modeling analysis, as well as the SEC behavior of the monomer and dimer of wt, S28N, and R121C variants, suggests that ANG dimerization occurs through the swapping of its N-termini and not through the swapping of the C-termini. Indeed, the C-terminal 3_10_-helix portion might represent a steric clash for accommodating a swapped C-terminus in the dimer, while this obstacle is absent with N-terminus swapping (Figure 6). Furthermore, the SEC patterns relative to wt, S28N, or R121C-ANG dimerization show a single symmetric dimeric peak. By contrast, RNase A and BS-RNase show a clearly visible shoulder in their dimeric peaks, suggesting that 3D-DS self-association occurs through the swapping of both N- and C-termini [40,55,56,63] (see Figure 3D). Furthermore, the proximity of the ANG-D to the monomer suggests that its structure is more compact than the known more extended RNase A C-swapped dimer [39]. That is, the SEC behavior of ANG-D resembles that of the N-swapped dimer of RNase A and onconase [41,56]. Moreover, the presence of a single dimeric peak is in accordance with ANG-D N-swapped nature, since more extensive self-association depends on the ability to swap more than one domain [64], and this actually occurs for pt-RNases [41,65].

Finally, we found that either the S28N or R121C mutations or also the enzyme dimerization affected only partially the enzymatic activity of both ANG variants vs. the fluorescent tetranucleotide used here; in particular, R121C-ANG is slightly more active than the wt (Table 1), which is in line with previous results [35]. However, all dimers display a reduced activity with respect to their monomers, suggesting that the two FUs cannot contemporarily exert their activity and that the ANG-D structure can somehow hinder the accommodation of the substrate.

## 4. Materials and Methods

### 4.1. Materials

RNase A, type XII-A, and yeast RNA were purchased from Sigma-Aldrich (Milan, Italy) and Boheringer (Ingelheim am Rhein, Germany), respectively. Acetic acid (HAc) and divinyl sulfone (DVS) were from Merck-Sigma. The cDNA of wt-ANG was kindly provided by Prof. E. Pizzo (The University of Naples Federico II, Naples, Italy). The fluorescent substrate 6-FAM (6-carboxyfluorescein)-tetranucleotide-BHQ^®^-1 (Black Hole Quencher-1), i.e., 6-FAM-dArU(dA)_2_-BHQ-1 was from Biomers.net, Ulm, Germany.

### 4.2. ANG Mutants Production

To introduce the mentioned mutations, the c-DNA of wt-ANG was processed using the following primers:

(i). H13A-ANG, F: 5′-CATTTCCTGACCCAGGCCTATGACGCTAAAC-3′, R: 5′-GTTTAGCGTCATAGGCCTGGGTCAGGAAATG-3′;
(ii). S28N-ANG, F: 5’-GGACGATCGTTACTGCGAAAACATTATGAGACGCCGTGGG-3′, R: 5′-CCCACGGCGTCTCATAATGTTTTCGCAGTAACGATCGTCC-3′;
(iii). R121C-ANG, F: 5′-GTCCATCTAGATCAGTCTATCTTCTGCAGGCCT-3′, R: 5′-AGGCCTGCAGAAGATAGACTGATCTAGATGGAC-3′.

The polymerase chain reaction (PCR) was carried out with the Phusion Hot Start II DNA Polymerase kit (Thermo-Fisher Scientific (Waltham, MA, USA)). The reaction mixtures contained 1 μL of plasmidic DNA, 10 μL of 5× buffer, 1 μL of 10 mM dNTPs mix, 1.25 μL of primers ([stock solution] = 25 μM), 0.5 μL polymerase, and 35.5 μL ddH_2_O. PCR cycles: 30” at 98 °C, 30 × cycles of 10” at 98 °C, 1 min at 57 °C, 5 min at 72 °C, then 12” at 72 °C. The amplification product was detected by electrophoresis with a 0.8% agarose gel with 1/10,000 SYBR^®^ dye solution and successively digested for 1 h with DpnI at 37 °C, in order to eliminate the original cDNA.

DH5α cells were incubated with 5 μL PCR product, transferred to an ice bath for 30 min, then to 42 °C for 20 min, and finally 2 min in the ice bath. Then, 250 μL of LB medium were added, and the sample was incubated at 37 °C for 1 h. Afterwards, 100 μL of *E. coli* bacteria were grown overnight (ON) on a Petri plate in 15 mL LB-agar containing 15 mg ampicillin (AMP). The colony whose sequenced DNA corresponded to the desired one was collected, amplified in 5 mL of LB medium containing 5 mg AMP, and grown at 37 °C. The plasmid was purified, sequenced, and used to transform BL21(DE3) cells that were in turn incubated with 100 ng of plasmidic DNA, transferred in ice bath for 30 min, incubated at 42 °C for 2 min and finally for another 2 min on ice. Then, 200 μL of LB were added, and the mixture was incubated at 37 °C for 1 h. Afterwards, 50 μL of this solution were transferred to a Petri plate containing 15 mL LB–agar and 15 mg AMP. The plate was kept ON at 37 °C, and a colony was collected, transferred in 5 mL LB containing 5 mg AMP, and grown ON at 37 °C. Subsequently, 500 μL of culture were mixed with glycerol and immediately frozen to −80 °C.

### 4.3. Protein Expression and Purification Analyses

Competent cells (BL21(DE3)) were grown for 6 h at 37 °C in 5 mL of TB medium and 0.5 mg AMP and then in 150 mL of the same broth, always ON at 37 °C. The inoculum was mixed with 0.1 g AMP in 1 L of TB at 37 °C. The protein production was induced with 0.4 mM IPTG when OD_600_ reached 2.4 and left ON with agitation at 37°C. Cell lysis was performed by suspending the pellet in 10 mL of 0.1 M Tris-acetate pH 8.4 plus 5 mM protease inhibitor phenylmethylsulfonyl fluoride (PMSF) and 2 g of lysozyme for 30 min. After this step, the pellet was solubilized, and the protein was extracted and refolded from inclusion bodies, as previously described for onconase^®^ [63]. The protein was further purified on a Superdex 75 HR 10/300 column equilibrated with 0.3 M NaCl + 0.1 M Tris-acetate, pH 8.4, and connected to an ÄKTA-FPLC system (GE-Healthcare, (Milan, Italy)). The protein was eluted at 0.3 mL/min flow rate, and its yield was spectrophotometrically determined at 280 nm with a Jasco V-650 spectrophotometer (Jasco Europe, Cremella (LC), Italy) with an $A_{280}^{0.1\%}$ = 0.834 calculated from the 12,500 M^−1^ cm^−1^ molar absorptivity [66]. The purified ANG variants were dialyzed against ddH_2_O (MilliQ, Merck Millipore, Billerica, MA, USA) and lyophilized as 0.5 or 1.0 mg aliquots.

Ten μg of the protein lyophilized from ddH_2_O were dissolved in 100 μL of 0.1% HCOOH and analyzed with a high-resolution mass spectrometer Xevo G2-S Q-TOF Instruments (Waters, Milford, MA, USA), in collaboration with the University of Padua. The mass vs. charge signals, visualized as BPI (base peak intensity) by the program MassLynx V4.1, were deconvoluted by the MaxEnt1 software to determine the average masses of the peaks. Other MS analyses were performed with a Bruker Ultraflextreme MALDI-TOF/TOF instrument of the “Centro Piattaforme Tecnologiche” (CPT), the University of Verona. A commercial sample of recombinant wt-ANG (Novoprotein, Summit, NJ, USA) was analyzed in parallel for comparison. Each ANG sample was suspended with an aqueous acetonitrile (ACN) solution (30:70 *v*/*v* ACN: 0.1% aqueous TFA) and incubated for 30 min at RT. The resulting solution was mixed at a 1:1 (*v*/*v*) ratio with the sinapinic acid (trans-3,5-dimethoxy-4-hydroxycinnamic acid) matrix (10 mg/mL in 1:1 ACN:H_2_O with 0.1% TFA). Then, 1 μL of the sample/matrix solution was spotted in triplicate onto a Ground Steel MALDI target plate (Bruker Daltonics, Billerica, MA, USA), allowed to dry at RT, and analyzed in the mentioned spectrometer. Mass calibration was performed with a protein standard mixture of myoglobin, cytochrome C, ubiquitin I, and insulin. Spectra were collected in the positive linear mode in the 5000 to 20,000 *m*/*z* range. Instrument settings: ion source 1: 19.93 kV; ion source 2: 18.77 kV; lens: 8.57 kV; delay time: 104 ns with a scale factor of 800; acceleration voltage: 20 kV; number of shots: 2000; in six different positions for one spectrum. All data were analyzed with the Flex Analysis Software (Bruker Daltonics).

### 4.4. Circular Dichroism (CD) Spectroscopy

The far and near UV-CD spectra of the ANG variants dissolved in 10 mM NaPi buffer, pH 6.7, were measured at 25 °C from 190 to 240 nm, and from 240 to 350 nm, respectively, using a Jasco J-710 spectropolarimeter (Jasco Europe, Cremella (LC), Italy). Protein concentration ranged from 0.3 to 1.0 mg/mL for near-UV and from 0.18 to 0.30 mg/mL for far-UV analyses. Spectra were normalized against concentration, and the far-UV signal was converted to molar ellipticity. Deconvolutions of far-UV CD spectra were performed in the 190–240 nm range, using the DichroWeb software online tools [67].

### 4.5. Induction of ANG Oligomerization

Oligomerization was induced by re-suspending the ddH_2_O-dialyzed powder in 40% aqueous HAc solution and lyophilizing it, following previously published protocols for RNase A [39,46].

The HAc lyophilized powder was solubilized in 0.40 M sodium phosphate (NaPi) buffer, pH 6.7 [40], and analyzed through size exclusion chromatography (SEC) by using a Superdex 75 Increase 10/300 GL column attached to the same ÄKTA-FPLC Purifier System (GE-Healthcare, Milan, Italy) equilibrated with the same 0.40 M NaPi buffer, pH 6.7. Each sample was eluted at 0.2–0.3 mL/min flow rate. In parallel, similar experiments were performed by (i) dissolving the HAc-lyophilized powder in ddH_2_O or (ii) dissolving the powder initially lyophilized from ddH_2_O in 0.40 M NaPi, pH 6.7. Subsequently, samples were kept in an ice bath before a SEC analysis performed under the same conditions mentioned above. The extent of ANG dimerization was calculated with the Unicorn 5.01 software (GE-Healthcare) by measuring the area ratio of each dimer or monomer peak vs. the total area of the peaks eluted.

### 4.6. Non-Denaturing Cathodic PAGE

In addition to SDS-PAGE, cathodic PAGE was performed under non-denaturing conditions. First, 15% acrylamide gels were electrophoresed at 200 V for 90 min in a tank containing 0.35% β-alanine brought to pH 4.0 in HAc buffer and immersed in an ice bath [68]. The purified species were desalted to 20 mM NaPi, and 5–6 μg of each were mixed with 4X methyl green and analyzed without boiling. The sample loading buffer (SLB) was composed of 0.04% methyl green dissolved in a 50% glycerol solution. Gels were stained with 0.1% Coomassie Brilliant Blue and destained with a 10% HAc/20% EtOH solution.

### 4.7. Cross-Linking with Divinyl Sulfone (DVS)

The dimers obtained from wt- or S28N-ANG were separately incubated with DVS, a short bifunctional cross-linker that can covalently connect the two active-site His residues of pt-RNases: one located near the N-terminus and the other located at the C-terminus [69]. Therefore, if dimerization took place through 3D-DS of N- or C-termini, or both, the DVS reaction should induce the formation of a covalent dimer, i.e., resistant against dissociation under the denaturing SDS-PAGE experimental conditions [48]. To do so, ANG dimers were brought to pH 5.0 at about 0.5–0.6 mg/mL concentration, in 0.10 M NaAc/HAc buffer, a 10% *v*/*v* DVS solution was added to a protein subunit:DVS final molar ratio of 1:1000, and incubation was performed at RT. Aliquots were withdrawn from the reaction at times ranging from zero to 96 h. Then, β-mercaptoethanol was added to each aliquot to quench the reaction. Finally, aliquots were analyzed with 15% polyacrylamide SDS-PAGE.

### 4.8. Enzymatic Activity Assays

ANG is definitely less enzymatically active than many pt-RNases. However, ANG activity can be tested on specific substrates, such as the dArU(dA)_2_ tetranucleotide derivatized with the 5′,6-carboxyfluorescein (6-FAM), and 3′,6-caroboxytetramethyl-rhodamine (6-TAMRA) fluorescent and quencher moieties, respectively, to form the 6-FAM-dArU(dA)_2_-6-TAMRA adducts [14,70]. In our case, 6-TAMRA was replaced by the Black Hole Quencher-1 (BHQ^®^-1) in the 6-FAM-dArU(dA)_2_-BHQ-1 substrate. Upon excitation at 485 nm, the 6-FAM terminus emits light with a λ_max_ = 535 nm, while BHQ-1 absorption spans from 400 to 700 nm, hence quenching the light emitted by 6-FAM. By contrast, the 6-FAM light emission can be observed following substrate cleavage. Assays were carried out at 25 ± 2 °C in black-framed 96-well plates in a 0.10 M MES/NaOH and 0.10 M NaCl buffer, pH 6.0, dissolved in RNase-free ddH_2_O (Merck-Millipore). We used 10 nM fluorogenic substrate, with WT- or S28N-ANG monomers or dimers at 0.5 to 1.0 µM in a final volume of 200 µL/well. The substrate concentration used is lower than the >1 mM K_m_ values estimated for ANG [18]. Therefore, as indicated in [54], the k_cat_/K_m_ ratio can be determined from Equation (1):

(1) kcatKm=ΔFΔtF∞−F0·E

with F_∞_ and F_0_ representing the maximum and initial fluorescence values registered, respectively, and [E] the molar concentration of each ANG or ANG-D variant used.

### 4.9. Molecular Modeling of the ANG Dimer

ANG-D was modeled using the homology modeling tool of the MAESTRO 12.2 software (Schrödinger). The X-ray structures of the RNase A N-swapped dimer (N_D_, pdb file 1A2W) and C-swapped dimer (C_D_, pdb file 1F0V) dimers [56,57] were used as templates to model the 1–119 aa structure of ANG-N_D_ and C_D_, respectively. The four 120-FRRP-123 C-terminal ANG residues were added to the ANG-N_D_ model, by means of the chimeric model generation tool of MAESTRO, using the ANG monomer structure (pdb code 1ANG) [8] as template. The ANG-N_D_ model and the RNase A N_D_ structure were refined by a step gradient energy minimization process applying the OPLS3e force field. Structures superimposition and image rendering were performed by PyMol v0.99 software (Schrödinger, Cambridge, MA, USA).

## 5. Conclusions

In the present work, we detected that human angiogenin can form a dimer, called ANG-D, upon HAc lyophilization. We also found that its two pathogenic variants S28N-ANG and R121C-ANG can dimerize as well and even at a higher extent than the wt. All species form ANG-D through the 3D-DS mechanism, and, either from the SEC behavior or by molecular modeling, we hypothesize that 3D-DS involves only the ANG N-terminus of the enzyme. All dimers retain enzymatic activity, even if slightly lower than the corresponding monomers. From this evidence, and considering the numerous variants involved in neurodegenerative diseases, as ALS and PD, this in vitro aggregation propensity shown by ANG can be considered important. In particular, if we take into account that the pathogenicity of S28N-ANG is ascribable to the loss of function related to a lack of Ser phosphorylation [36] that may in turn favor the ANG inactivation by the cellular ribonuclease inhibitor [71,72], also dimerization might be a factor limiting the physiological role of ANG. Indeed, dimerization could interfere with the entry of unphosphorylated ANG into the nucleus, in this way negatively affecting its fundamental biological actions [73]. In addition, the R121C mutation could attenuate ANG’s physiological role upon dimerization that we detected as being able to induce a decrease in the enzymatic activity.

In conclusion, it could be worth evaluating in the future if ANG self-association, although not being massive, might affect residues or domains crucial for the intracellular routing of the protein, and/or affect for instance ANG action in the stress granules [32]. This scenario might, in fact, help the onset and/or development of the aforementioned neurodegenerative diseases.

## Figures and Tables

**Table 1:** MW of the ANG variants, percentages of M and D formed, and enzyme activity values.

ANG Species	WT	WT-D	S28N	S28N-D	R121C	R121C-D	H13A
Properties							
Yield% *	89.4 ± 1.2	10.1 ± 1.0	84.3 ± 1.0	14.7 ± 0.9	80.3 ± 1.6	18.0 ± 1.2	≈100
**Theoretical MW**	14,121	28,242	14,148	28,296	14,068	28,136	14,055
**Mass Spec MW**	14,141.0	---	14,148.2	---	14,070		14,054.9
**SEC-Elut Vol (mL)**	15.26 ± 0.05	13.60 ± 0.06	15.30 ± 0.08	13.62 ± 0.11	15.24 ± 0.05	13.45 ± 0.09	15.29 ± 0.04
Calc. MW (SEC) **	8985	17,090	8960	17,125	8992	17,245	8965
**Ratios M/M-SEC** **D/D-SEC**	1.57	1.65	1.58	1.65	1.56	1.63	1.58
**Ratio D-SEC/M-SEC**	---	1.90	---	1.91	---	1.92	1
K_cat_/k_m_(10^3^ M^−1^s^−1^) ***	1.75 ± 0.31	1.02 ± 0.28	1.28 ± 0.23	0.87 ± 0.29	1.96 ± 0.26	1.26 ± 0.20	---

* The sum of the yields of M + D is not 100% because traces of larger species were produced by all species. In particular: WT 0.5%; S28N 1.0%; R121C 1.7%. ** Calculated from the calibration curve reported in the Supplementary Figure S2. *** Calculated from Equation (1) [54]. “---“ means “not applicable”.

## Supplementary Materials

The following are available online at https://www.mdpi.com/article/10.3390/ijms221810068/s1.
