# Supplementary material for: Dimerization of Human Angiogenin and of Variants Involved in Neurodegenerative Diseases
Source: Int J Mol Sci. 2021 Sep 17;22(18):10068. doi: 10.3390/ijms221810068 (PMC8468037; doi:10.3390/ijms221810068)
Supplement: Supplementary file 1 [file ijms-22-10068-s001.zip › ijms-1348935-supplementary.pdf]

Supplementary data for

Dimerization of Human Angiogenin and of Variants Involved in Neurodegenerative Diseases

by

S. Fasoli[1#], I. Bettin[1#], R. Montioli[1], A. Fagagnini[1,3], D. Peterle[2], D.V. Laurents[3], G. Gotte[1*]

[*]Corresponding Author

[#]Contributed equally

The data included are Supplementary Figures S1-S5

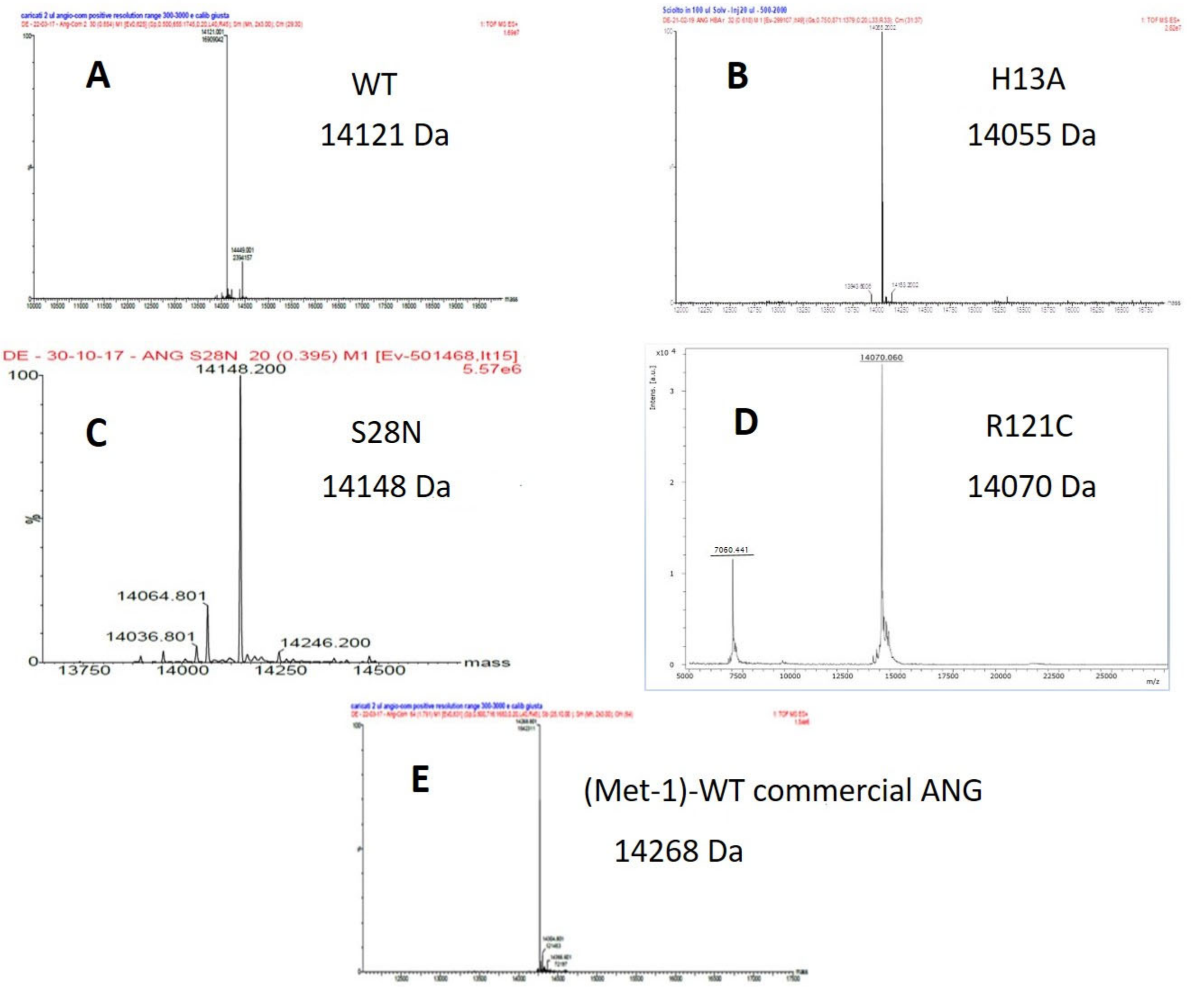

Figure S1. Mass Spectra of the ANG variants produced. **(A)** wt-ANG; **(B), (C)** and **(D)** the three H13A, S28N and R121C-ANG variants. **(E)** MS analysis of commercial recombinant wt-ANG used as standard in which the Met(-1) has not been cleaved as reported by the manufacturer, justifying the higher MW value with respect to all ANG variants (wt and mutants) produced and processed with aminopeptidase in our labs. Experimental details are reported in the Mat & Meth. Section.

Superdex 75 HR 10/300 Increase SEC column

STD proteins	MW (kDa)	Log MW	Elut. Vol. (ml)
Alcohol Dehydrogen.	150	5.176	8.54
Bovine SerAlbumin	66	4.820	9.72
Ovalbumin	45	4.653	10.84
Carbonic Anhydrase	29	4.462	12.18
RNase A	13.7	4,136	14.24
Cytochrome C	12.4	4.093	14.48

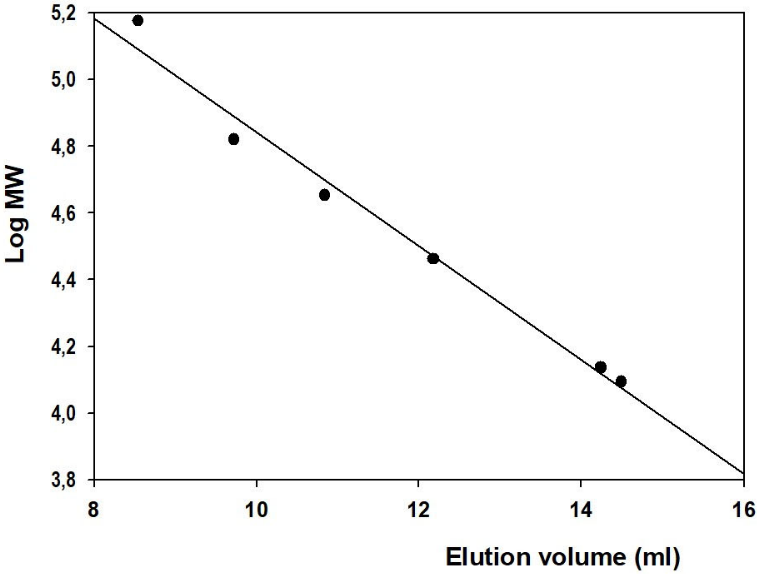

calibration curve

Figure S2. Calibration curve of the SEC Superdex 75 HR10/300 Increase column. About 200 to 500 µg of the various MW-standard proteins that are mentioned in the table on the right have been injected in the column equilibrated with 0.40 M NaPi, pH 6.7, and eluted at 0.20-0.25 ml/min, like for the ANG species analysed.

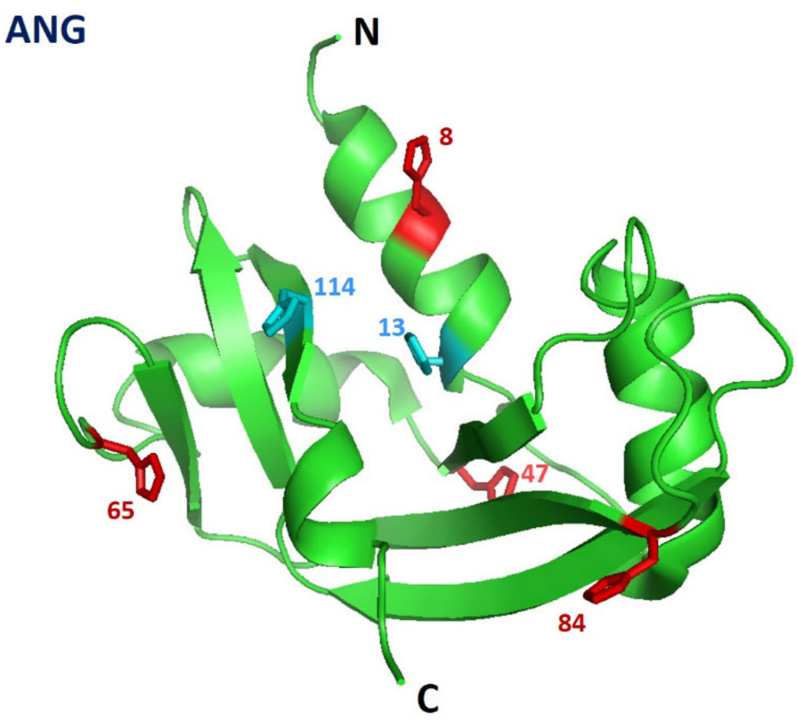

Figure S3. 3D structure of wt-ANG with His residues highlighted. The cartoon has been built with the pdb 1ANG file using the PyMol software, indicating the position of both N- and C-termini. The two His residues belonging to the active site (13 and 114) are visible in cyan, all the other His residues of ANG are numbered and coloured in red.

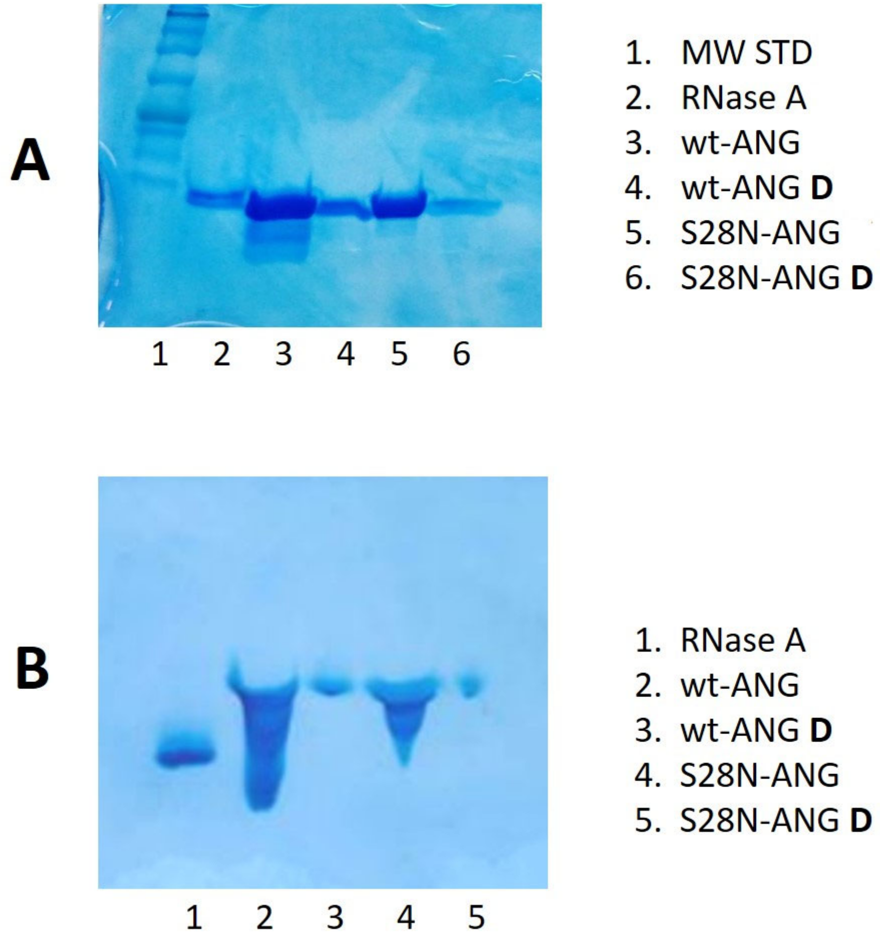

Figure S4. Electrophoresis of ANG and ANG-D of wt and S28N mutant. (A) 15% acrylamide SDS-PAGE of the ANG species. Gel was run for 90 min at 200 V. All the ANG species show the same electrophoretic mobility, suggesting that the denaturing conditions induce the dissociation of the dimers to monomer. **(B)** 15% acrylamide cathodic PAGE. Gel was electrophoresed at 200 V for 90 min in a tank containing 0.35% β-alanine brought to pH 4.0 with HAc, and immersed in an ice-bath [47]. Both monomeric ANG species showed a "drag" behaviour, absent in RNase A (lane 1). Experiments performed with different acrylamide % and different voltage conditions (not shown) did not afford a better separation of ANG monomer and dimer species.

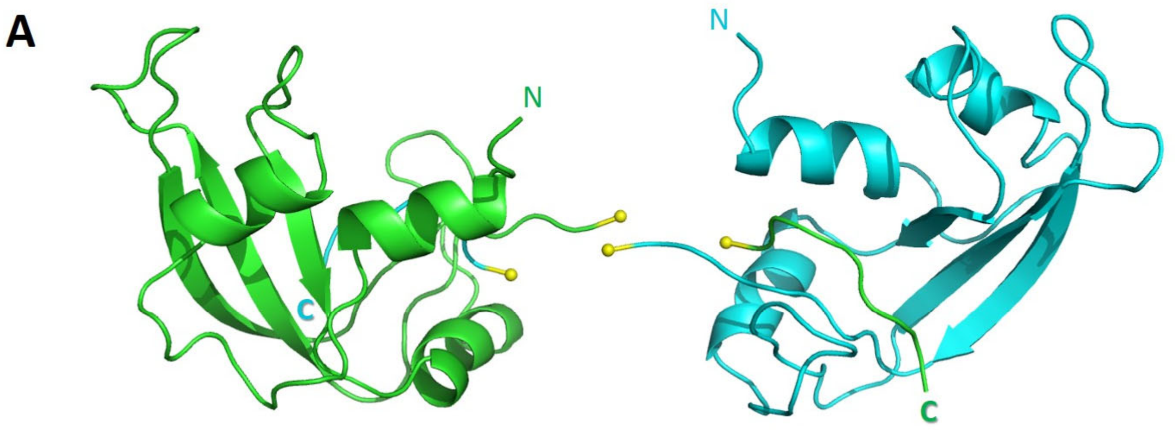

B

```
RNAse A         QKNVACKNGQTNCYQSYSTMSITDCRETGSSKYPNCAYKTTQANKHIIVACEGNPYVPVH    119
Angiogenin      NKNGNPH--RENLRISKSSFQVTTCKLHGGSPWPPCQYRATAGFRNVVVACENG--LPVH    114
                :**   :  : *   * *::.:* *:  *.* :* * *::* . ::::****..  :***

RNAse A         FDASV----       124
Angiogenin      LDQSIFRRP       123
                :* *:
```

Figure S5. 3D modelling investigation for a possible ANG C-swapped dimer. **(A)** Cartoon representation of the ANG-C_D model in which the two protomers are coloured in green and cyan, respectively, and swap their C-termini. Interruption points are highlighed as yellow dots and the corresponding aa segment is indicated in **(B)** in the sequence alignment of the C-terminal region of ANG and RNase A. The model image was rendered by PyMol software (Schrödinger) and the multiple sequence alignment was generated by Clustal Omega Tool (EMBL-EBI).
